# Supplementary material for: Do black lives matter to employers? A combined field and natural experiment of racially disparate hiring practices in the wake of protests against police violence and racial oppression
Source: PLoS One. 2022 May 25;17(5):e0267889. doi: 10.1371/journal.pone.0267889 (PMC9132270; doi:10.1371/journal.pone.0267889)
Supplement: S1 File — (PDF) [file pone.0267889.s001.pdf]

Do black lives matter to employers?
A combined field and natural experiment of racially disparate hiring practices in the wake of protests against police violence and racial oppression

Supporting Information: Further Methodological Details

In this document, we present further methodological details about our study as well as the robustness tests noted in the main text of the paper.

Selection of Names

The extent to which the names we used for the fictitious applicants in our experiment solely signal race rather than additional characteristics such as socioeconomic status is an important consideration for assessing the validity of our inferences about racial discrimination [1, 2]. Therefore, we devote space here to describing how we came to use the particular names noted in the main text. Our selection of names was guided by prior methodological work on audit studies, including work by Gaddis [1]. We chose names with a high degree of racial distinctiveness, and which have a similar level of socioeconomic background relative to the other names used. For instance, using New York state birth record data from 1994 to 2012, Gaddis conducted a survey experiment over the course of 12-months, using a sample from individuals from MTurk to examine the racial distinctiveness of commonly used first names [1]. He asked respondents to state the race or ethnicity of a randomized set of first names. For the names used in the present study, he found the following:

- "Black" First Names: Jabari (92.1% of respondents perceived this name to be from a Black individual), Tremayne (86.4% Black), Darnell (80.1% Black), Shanice (85.9% Black), Erykah (74.1% Black), and Janae (76.6% Black).

- “White” First Names: Ethan (72.6% of respondents perceived this name to be from a White individual), Ryan (82.2% White), Jake (93% White), Claire (86.4% White), Emily (86% White), and Katelyn (85.9% White).

We sought to select names across race with a similar degree of socioeconomic status, as suggested by first names. In his study, Gaddis used demographic and state birth records data to examine mothers’ average educational attainment among individuals with common first names [1]. Average maternal education in New York State for individuals with the following names, born between 1994 and 2012, is as follows:

- “Black” First Names: Jabari (56.8% of mothers with some college education completed), Tremayne (34.1%), Darnell (28.3%), Shanice (24.9%), Erykah (48.1%), and Janae (50.6%).
- “White” First Names: Ethan (70.4% of mothers with some college education completed), Ryan (69.4%), Jake (78.2%), Claire (89.2%), Emily (64.3%), and Katelyn (61.8%).

For the sake of comparison, note that according to the 2000 U.S. Census, 54.1% of the White population aged 25 or older had some college education, compared to 42.5% of the Black population.

For last names, we again attempted to choose names with a high degree of racial distinctiveness. As reported in the study by Gaddis [1], the racial distribution in the total U.S. population per the 2000 U.S. Census for each of the last names used in this study is as follows:

- “Black” last names: Booker (65.6% Black), Jackson (53% Black), Jefferson (75.2% Black), Mosley (52.8% Black), Washington (89.9% Black);

- “White” last names: Becker (96.4% White), Decker (95.4% White), Hartman (95.4% White), McGrath (95.9% White), Meyer (96.1% White), Walsh (95.9% White).

In summary, two potential limitations of our study are that the names do not strongly enough signal a given race, and that the names signal more characteristics of the individual than race, thereby hindering our efforts to isolate the particular effect of applicant race on employer responses. In our view, the second limitation is the more likely of the two. Nevertheless, we would remind the reader that our interest in this study is on comparing the racial gap in employer responses. Because we used the same names at each wave of the study, presumably the proportion of the gap in employer responses due to race versus some other characteristics such as socioeconomic status would remain largely the same across waves. In short, because we are making cross-wave comparisons, the fact that our names may be signaling more than just race may be less of a problem than if we were focused on a single wave or within-wave analysis.

Prior Profession

As noted in the main text, we constructed the fictitious résumés so that an applicant’s most recent profession was as a police officer, firefighter, or code enforcement officer, with approximately three years of experience in these professions. Relevant to our design is the fact that policing is a profession with a high rate of turnover, with some estimates suggesting that nearly eight percent of police officers voluntarily resign from the job each year (i.e., excluding retirements) [3]. Low morale, stress, and disillusionment with the job, along with injuries, difficult supervisors, and irregular schedules, are commonly cited reasons for voluntarily leaving the profession before retirement [4]. Hence, it is not uncommon for former police officers to seek

new employment and new career paths. Resignations were especially acute following the protests against police violence in the wake of George Floyd's murder, although the net decrease in the number of police officers nationwide between 2019 and 2020 was minimal, presumably because departures were offset by new hires [5, 6].

Research Ethics

This study was approved by our university's institutional ethics committee. As noted in the main text, by design correspondence studies of discrimination involve deception, given that participants, employers in our case, are not made aware that they are participating in an experiment. Therefore, we did not obtain informed consent from the employers who participated in the study. There are trade-offs between the costs and benefits of audit and correspondence designs, but the use of deceptive study designs may be acceptable under the following conditions: a) other research designs (i.e., that do not use deception) cannot similarly overcome the methodological obstacles to measuring discrimination; b) the topic has social relevance; and c) there is minimal harm to participants and minimal negative externalities [7-9].

Regarding the first condition, a common justification for deception is that correspondence experiments offer crucial methodological advantages for detecting discrimination [8]. For instance, observational studies are typically hampered by the possibility of omitted variables and selection bias, thereby undermining a researcher's ability to determine if certain groups are discriminated against in the labor market. Surveys of employers designed to query about discriminatory attitudes and behavior may be plagued quite substantially by social desirability bias, if employers overstate their willingness to hire members of a subordinate group. Carefully designed field experiments constitute a robust research design for measuring discriminatory

behavior and for assessing how such behavior changes over time due to events or intervention (e.g., equal opportunity legislation).

On the second condition, field experiments can provide convincing evidence of the extent of discriminatory behavior that cannot be obtained in an unbiased way by alternative methods [9]. This information can be crucial for assessing progress towards a more equitable society and for designing equitable employment practices and employment legislation.

The third condition of minimal harm can be achieved by designing experiments that minimize inconveniences and time burdens on participants, and that do not have unacceptable negative externalities. We took several steps in our study to reduce the possibilities of harm to our participants (i.e., the employers) or negative externalities to other job applicants. For instance, we confirmed through a review of Manpower's annual Talent Shortage Survey that we were targeting occupations in industries that commonly had abundant job openings [10]. In fact, Manpower's survey revealed that our four targeted occupations (skilled trades, drivers, sales, office and customer support) are among the occupations with the greatest levels of labor shortage in the country (others include teachers and healthcare professionals). We would suggest that the regular demand for applicants in these occupations helped minimize the risk that our fictitious applicants would deprive a real applicant of a job.

As for the time burden on employers, we are cognizant that the time to review a résumé and respond to a fictitious applicant is not negligible. To help reduce the time burden on the part of sampled employers, we only applied to one job opening per employer even if they had multiple openings over our data collection period. We also sought to promptly reply to correspondence from employers, declining offers to interview for a job.

SI References

1. Gaddis SM. How black are Lakisha and Jamal? Racial perceptions from names used in correspondence audit studies. Sociol Sci. 2017;4: 469–489.
2. Landgrave M, Weller N. Do name-based treatments violate information equivalence? Evidence from a correspondence audit experiment. Polit Anal. 2022;30: 142–148.
3. Wareham J, Smith BW, Lambert EG. Rates and patterns of law enforcement turnover: A research note. Crim Justice Policy Rev 2015;26(4): 345–370.
4. Hilal S, Litsey B. Reducing police turnover: Recommendations for the law enforcement agency. Intl J Police Sci & Manag 2020;22(1): 73–83.
5. Mourtgos SM, Adams IT, Nix J. Elevated police turnover following the summer of George Floyd protests: A synthetic control study. Criminol Public Policy 2022;22(1): 9–33.
6. Li W, Mahajan I. Police Say Demoralized Officers Are Quitting In Droves. Labor Data Says No. The Marshall Project. 2021 Sep 1 [cited 2022 Apr 3]. Available from: https://www.themarshallproject.org/2021/09/01/police-say-demoralized-officers-are-quitting-in-droves-labor-data-says-no.
7. Zittel T, Louwerse T, Pedersen HH, Schakel W. Should we conduct correspondence study field experiments with political elites? Intl Polit Sci Rev. 2021. doi: 10.1177/01925121211026489.
8. Riach PA, Rich J. Field experiments of discrimination in the market place. Econ J. 2002;112(483): 480–518.
9. Pager D. The use of field experiments for studies of employment discrimination: Contributions, critiques, and directions for the future. Ann Am Acad Pol Soc Sci. 2007;609(1): 104–133.
10. Manpower Group. *2018 Talent Shortage Survey United States*. 2018 [Cited 7 May 2019]. Available from: https://cdn2.hubspot.net/hubfs/2942250/Local%20Infographics/2018_TSS_Infographics-United%20States.pdf.

S1 Table. Logit models of employer responsiveness to job applications, by time period and applicant race.

	Model 1			Model 2			Model 3		
	Coef	(SE)		Coef	(SE)		Coef	(SE)	
Constant	-1.522	(0.137)	***	-0.898	(0.447)	***	-0.859	(0.451)	***
Race (White)	0.397	(0.182)	*	0.386	(0.189)	*	0.298	(0.211)	
Time Period 2 (Post-Floyd)	0.505	(0.251)	*	0.352	(0.321)		0.336	(0.343)	
Race x Time Period 2	-0.647	(0.351)	+	-0.676	(0.359)	+	-0.685	(0.395)	+
Gender (Male)				0.145	(0.159)		0.148	(0.159)	
Boston (vs. Philadelphia)				0.231	(0.183)		0.230	(0.183)	
Craigslist (vs. Indeed.com)				-0.131	(0.216)		-0.130	(0.217)	
Office/Cust. Support Job (vs. Driver)				-0.725	(0.224)	***	-0.729	(0.224)	***
Sales Job (vs. Driver)				-0.189	(0.215)		-0.187	(0.216)	
Skilled Trades Job (vs. Driver)				-1.079	(0.272)	***	-1.089	(0.273)	***
Firefigher (vs. Police)				0.104	(0.108)		0.082	(0.191)	
Code Enf. (vs. Police)				-0.050	(0.118)		-0.279	(0.243)	
Race x Firefighter							0.018	(0.263)	
Race x Code Enf.							0.364	(0.293)	
Time Period 2 x Firefighter							-0.298	(0.386)	
Time Period 2 x Code Enf.							0.347	(0.388)	
Race x Time Period 2 x Firefighter							0.569	(0.471)	
Race x Time Period 2 x Code Enf.							-0.612	(0.547)	
Month indicator included	NO			YES			YES		
Day of week indicator included	NO			YES			YES		

+ $p<0.10$, * $p<0.05$, ** $p<0.01$, *** $p<0.001$ (two-tailed test).
N = 1,634 job applications (817 jobs). Standard errors clustered by job.
To condense the presentation of results, we have omitted from the table the coefficients and standard errors for the indicators of the month and day of the week when job applications were submitted.
