# Supplementary material for: Do black lives matter to employers? A combined field and natural experiment of racially disparate hiring practices in the wake of protests against police violence and racial oppression
Source: PLoS One. 2022 May 25;17(5):e0267889. doi: 10.1371/journal.pone.0267889 (PMC9132270; doi:10.1371/journal.pone.0267889)
Supplement: Attachment — Submitted filename: ResponsetoReviewers.docx [file pone.0267889.s002.docx]

**Do black lives matter to employers?**

**A combined field and natural experiment of racially disparate hiring practices in the wake of protests against police violence and racial oppression**

**PLOS ONE Manuscript: PONE-D-22-02153**

**Response to the Editor and the Reviewers**

We thank the reviewers and editor for their many helpful and insightful comments. We gave careful attention to all of the detailed suggestions offered by the reviewers and we believe we have made corresponding revisions in the paper to address them. Below we reproduce reviewer comments in italics (with the exclusion of general summary comments), with our response immediately thereafter. Line numbers referenced below refer to those found in the unmarked version of the revised manuscript.

**Response to Reviewer #1:**

Reviewer 1 recommended an acceptance. We appreciate the enthusiasm for our work.

**Response to Reviewer #2:**

*I definitely think that this paper merits publication. It is an original contribution to a field of research on its way to get “saturated” and the study is carefully executed, including convincing arguments for the choice of names, occupations, and the fictitious applicants’ prior professions. The analyses are also well done and presented in an accessible way. Still, I would recommend a bit more reflection on how some of the authors’ choices may affect their findings, as well as pointing out some alternative interpretations of the results that should be discussed. The authors have made an interesting choice in letting the fictitious applicants have former work experience as police officers, firefighters, or code enforcement officers. In the supporting information document, they argue convincingly that it is not uncommon for individuals with such work experience to seek new career paths. However, the special type of prior professions begs the question of whether one would find the same effects for applicants with completely different qualifications and work experiences, or whether the change in employer behavior would be similar across different backgrounds. In other words: do the authors interpret their findings as a general reduction in hiring discrimination against black or primarily a reduction in discrimination against individuals with prior experience as police officers and fire fighters?*

Reviewer 2 has hit upon a central question. We do not wish to extrapolate beyond what is sensible with our data, so all we can reasonably say at the moment is that our findings pertain to the front-line public sector employees that are the focus of our study. However, in the Discussion section of the paper, we call for additional research using different qualifications than the ones used in this study (lines 462-470). Nevertheless, group threat theory leads us to expect that there would be a general reduction in employment discrimination in the wake of highly publicized police violence and associated protest activity.

*Relatedly, the authors point out on p. 15 that the “results may not fully generalize to the population of jobs in a labor market.” This is an understatement. All field experiments suffer from this limitation, but this one most certainly so as the four different categories of service-related jobs that are applied to represent a very small set of available jobs. I would strongly recommend that the authors acknowledge this limitation and offer some more space in reflecting on its implications.*

We thank the reviewer for the suggestion to consider the implications of our sampling design with respect to generalizability and study limitations. We have added some corresponding discussion to lines 335-346.

*The authors also point out that the general labor market situation characterizing the two waves was quite different because the first wave was conducted in the midst of the pandemic when the unemployment rates increased dramatically. However, I do not find that they really reflect on this problem besides arguing that a focus on black/white differences in callbacks is the most relevant outcome measure in both waves. Now, I certainly do not think that the reversal of the black/white disparities could be explained by a change in general employment levels, but some previous studies (e.g., the work of Stijn Bart) suggest that minority disadvantage tend to be higher when unemployment rates are high. Indeed, it would be beneficial if the authors added information about the average black/white employment rates in the two periods and in the areas of which the study was conducted. If the black/white difference was reduced, this would probably explain some of the effect, yet the gap was probably not turned on its head, suggesting that the study actually do document a reduction in discrimination.*

We appreciate the suggestion to reflect on the likely differences in discrimination rates in tight vs. slack labor markets. We now do so on lines 317-334. Given that discrimination tends to be greater in slack labor markets, our findings may actually understate the effect of highly publicized police violence and widespread protest activity, if it had occurred at a time not characterized by high unemployment.

*The authors could also consider comparing the discrimination rates in the two separate waves to the overall discrimination rate against black applicants across time in the cited Quillian et al. study. I believe the readers would be interested in learning whether the baseline results (wave 1) align with what has already been established.*

We concur that it is useful to compare baseline rates of discrimination in our study to the trends examined in the Quillian et al. (2017) meta-analysis. Indeed, we find that the ratio of White-to-Black call-backs in our study are highly similar to those reported in the Quillian et al. study (see lines 383-391).

*Finally, I think the paper would benefit from a table with a description of study characteristics. This information provides a useful overview of the study an makes it more transparent.*

We have added Table 1 showing study characteristics, namely observations by time period, applicant race, gender, and prior profession.

**Response to Reviewer #3:**

*1. It would be helpful to the reader to understand how it is that the authors came to be doing a study that was interrupted by the George Floyd protest. This currently reads almost as if this was by design, but obviously this can’t be the case. What was the larger purpose of the study?*

Reviewer 3 suggested we provide further detail about the original motivation of our study, which was interrupted by the murder of George Floyd. Our original study was in fact designed to examine reactions to police officers and their potential stigma in the labor market. Of course we could not have anticipated the murder of Mr. Floyd or the reaction to his death. Whereas the attention generated by the murder of Mr. Floyd was a vast order of magnitude greater than many deaths, the reviewer presumably knows that roughly 1,000 to 1,100 individuals are killed by the police each year in the United States (according to a variety of data sources, including the Washington Post police shootings database, Fatal Encounters, and mappingpoliceviolence.org). Hence, while our original intent was to study reactions to the police in the context of what might be called their more typical incidents of violence, the death of Mr. Floyd tragically provided an opportunity to examine our question in the context of widespread protests and media attention. We have sought to clarify these points on lines 170-176.

*2. Consider how the use of a former police officer as a characteristic of the candidate following the case of police killing limits the generalizability of the study. For example, might it be the case that a former white police officer is particularly punished in this circumstance? The interaction models speak to this, I believe, but the authors should be more explicit about this.*

Per the reviewer’s excellent suggestion, we provide a broader discussion of the limitations of our design choices (lines 347-355), including limits on generalizing beyond the professional background characteristics examined in the study. In the Discussion section, we encourage future research that is designed in such a way to provide broader generalizability (lines 462-470).

*3. The point about the study not generalizing to the entire labor market is true, but probably doesn’t need to be said. Does any audit study generalize to the entire labor market?*

While appreciate the reviewers point, but have opted to retain this particular discussion about generalizability in order to address Reviewer 2’s comments.
